# Lower abdominal and pelvic radiation and testicular germ cell tumor risk

**DOI:** 10.1371/journal.pone.0239321

**Published:** 2020-11-11

**Authors:** Kevin T. Nead, Nandita Mitra, Benita Weathers, Louisa Pyle, Nnadozie Emechebe, Donna A. Pucci, Linda A. Jacobs, David J. Vaughn, Katherine L. Nathanson, Peter A. Kanetsky

**Affiliations:** 1 Department of Radiation Oncology, Perelman School of Medicine at the University of Pennsylvania, Philadelphia, Pennsylvania, United States of America; 2 Department of Epidemiology, Department of Radiation Oncology, MD Anderson Cancer Center, Houston, Texas, United States of America; 3 Department of Biostatistics, Epidemiology and Informatics, Perelman School of Medicine at the University of Pennsylvania, Philadelphia, Pennsylvania, United States of America; 4 Division of Translational Medicine and Human Genetics, Department of Medicine, Perelman School of Medicine at the University of Pennsylvania, Philadelphia, Pennsylvania, United States of America; 5 Division of Human Genetics and Metabolism, Children’s Hospital of Philadelphia, Philadelphia, Pennsylvania, United States of America; 6 Department of Cancer Epidemiology, H. Lee Moffitt Cancer Center and Research Institute, Tampa, Florida, United States of America; 7 Department of Epidemiology and Biostatistics, College of Public Health, University of South Florida, Tampa, Florida, United States of America; 8 Division of Hematology-Oncology, Department of Medicine, Perelman School of Medicine at the University of Pennsylvania, Philadelphia, Pennsylvania, United States of America; 9 Abramson Cancer Center, Perelman School of Medicine at the University of Pennsylvania, Philadelphia, Pennsylvania, United States of America; University of Zurich, SWITZERLAND

## Abstract

**Background:**

Testicular germ cell tumor (TGCT) incidence has increased in recent decades along with the use and dose of diagnostic radiation. Here we examine the association between reported exposure to diagnostic radiation and TGCT risk.

**Methods:**

We conducted a case-control study of men with and without TGCT recruited from hospital- and population-based settings. Participants reported on exposures to 1) x-ray or CT below the waist and 2) lower GI series or barium enema, which consists of a series of x-rays of the colon. We also derived a combined measure of exposure. We used logistic regression to determine the risk of developing TGCT according to categories of exposures (0, 1–2, or ≥3 exposures) and age at first exposure, adjusting for age, year of birth, race, county, body mass index at diagnosis, family history of TGCT, and personal history of cryptorchidism.

**Results:**

There were 315 men with TGCT and 931 men without TGCT in our study. Compared to no exposures, risk of TGCT was significantly elevated among those reporting at least three exposures to x-ray or CT (OR_≥3 exposures_, 1.78; 95% CI, 1.15–2.76; p = 0.010), lower GI series or barium enema (OR_≥3 exposures_, 4.58; 95% CI, 2.39–8.76; p<0.001), and the combined exposure variable (OR_≥3 exposures_, 1.59; 95% CI, 1.05–2.42; p = 0.029). The risk of TGCT was elevated for those exposed to diagnostic radiation at age 0–10 years, compared to those first exposed at age 18 years or later, although this association did not reach statistical significance (OR, 2.00; 95% CI, 0.91–4.42; p = 0.086).

**Conclusions:**

Exposure to diagnostic radiation below the waist may increase TGCT risk. If these results are validated, efforts to reduce diagnostic radiation doses to the testes should be prioritized.

## Introduction

Testicular germ cell tumor (TGCT) is the most common cancer in white men aged 15–44 years of age. The incidence of TGCT has increased rapidly in recent decades particularly among individuals of European ancestry [1]. Although multiple hypotheses investigating the impact of environmental exposures on risk of TGCT have been proposed, few have been found to be robustly associated with this increasing incidence [2]. One factor contributing to the rise in TGCT incidence may be the 20-fold increased use of diagnostic radiation in recent decades [3]. During this same period the mean effective dose of diagnostic radiation, a measure of how dangerous an individual’s exposure to radiation can be, has increased seven-fold [4].

The risk of cancer secondary to diagnostic radiation traditionally has been modeled based on extrapolation from observations in atomic bomb survivors. This cohort has long been considered the standard for the quantitative assessment of carcinogenesis secondary to low dose diagnostic radiation exposure due to the large number of individuals with exposure in the diagnostic radiation range (5–100 mSv) and the collection of cancer incidence and mortality data over a greater than 70 year period [5]. These data suggest that radiation doses as low as 5–10 mSv may lead to measurable increases in the risk of solid malignancy at the population level [6]. This exposure threshold is currently and historically less than the average computed tomography (CT) of the abdomen or pelvis (~6–8 mSv), or barium enema, a diagnostic procedure that includes a series of x-rays to the colon also known as a lower GI series (~6–8 mSv) [3, 7, 8]. Moreover, doses received from individual scans can significantly exceed reported averages. Up to a quarter of children who have a single CT of the abdomen and pelvis may receive a dose of 20 mSv or higher [9], although these doses may be less common with the initiation of the Image Gently campaign [10]. Radiation exposure in children is of particular concern as the risk of cancer may be an order of magnitude higher than in adults [6].

More recently, direct epidemiologic evidence supports an association between diagnostic radiation and risk of several types of cancer. Women undergoing frequent x-rays for scoliosis monitoring may have an increased risk of breast cancer [11, 12], and individuals undergoing repeat routine dental x-rays may have an increased risk of thyroid cancer [13]. A large National Health Service study among 175,000 children, showed an excess risk of subsequent brain tumors and leukemia attributed to CT scans [14]. Total-dose and number of exposures were associated with increasing cancer risk in each of these studies [11–14].

Whether an association between exposure to diagnostic radiation and elevated TGCT risk exists is currently unknown. Although testicular dose during diagnostic radiation can be reduced significantly with testicular shielding [15], audits of utilization in children show that appropriate use and positioning occurs in only 25% of diagnostic scans [16]. In the absence of shielding, diagnostic radiation doses to the testes can exceed 20mSv [15]. Therefore, if diagnostic radiation is shown to confer an elevated TGCT risk a readily available intervention exists. In this study we utilize information from a case-control study of TGCT to examine the association between reported exposure to diagnostic radiation below the waist and TGCT risk. We hypothesize that exposure to diagnostic radiation increases TGCT risk, and that this risk is greater with a larger number of exposures and an earlier age at first exposure.

## Materials and methods

### Case subjects

Between September 2007 and May 2013, we used a hybrid strategy incorporating both a clinic-based and a population-based approach to enroll men between the ages of 18 and 55 with an incident diagnosis of TGCT who resided in one of nine Pennsylvania counties (Berks, Bucks, Chester, Delaware, Lancaster, Lehigh, Montgomery, Northampton, and Philadelphia). From May 2013 to December 2015, recruitment of men with TGCT continued using only a clinic-based approach. Because of the high (95%) 5-year survival for TGCT [17] we defined an incident diagnosis as that within two years of study enrollment.

For clinic-based recruitment, men with TGCT who were seen at the Genitourinary Oncology clinic at the Hospital of the University of Pennsylvania were approached while at their clinic appointment, invited to participate in the study and given the study questionnaire and informed consent form. For population-based recruitment, men diagnosed with TGCT within the five-year span of 2006 to 2010 were enumerated through the Pennsylvania State Cancer Registry (PACR). We attempted to contact via mail all men with TGCT identified in the PACR who met age, residence, and incident criteria and who were not approached at the Hospital of the University of Pennsylvania. These men were mailed a packet that contained an introductory letter that briefly described the study, the study questionnaire, and an informed consent form. This study was approved by the Institutional Review Board of the University of Pennsylvania, and all participants provided written informed consent.

Information on tumor subtype was obtained through medical record review for participants recruited in clinic and from the PACR for participants recruited by mail. Case subjects were classified as having a seminomatous, non-seminomatous, or mixed (regions of both seminoma and non-seminoma) TGCT based on histological diagnosis. Regardless of recruitment strategy, all case subjects were asked to complete a self-administered questionnaire that elicited information on known and presumptive risk factors for TGCT (and subsequently to provide a biospecimen for genotyping). A copy of the study questionnaire for men with TGCT can be found in the Supplemental materials. All data were entered into an in-house web-based database management system.

We enrolled a total of 318 men with TGCT who met age, residency, and incidence criteria. Between September 2007 and May 2013, we identified 702 men with TGCT from the PACR. We recruited 238 of these men into our study: 100 were enrolled at the Genitourinary Oncology clinic at the Hospital of the University of Pennsylvania and 138 were enrolled using mailer packets. Between May 2013 to December 2015, we enrolled an additional 80 men with TGCT from the Genitourinary Oncology clinic at the Hospital of the University of Pennsylvania, a setting from which we previously and currently enroll 97% of eligible men approached to TGCT research protocols. We removed three men from our analysis who subsequently withdrew from the study for a final analytic dataset of 315 men with TGCT.

### Control subjects

Men without TGCT were contacted for enrollment from January 2009 to June 2012 through a combination of random-digit dialing (frequency-matched by age and race to men with TGCT) and address based sampling (frequency-matched by geographical location to men with TGCT). Details of control recruitment, response rates, and assessment of selection bias have been previously published [18].

Briefly, men without TGCT were originally recruited via random-digit dialing from counties in proximity to Philadelphia, Pennsylvania, from which TGCT cases had been previously identified. Screening was conducted and eligible respondents were sent the research study questionnaire, consent form, and a saliva collection kit. There were 44,599 attempted contacts with 343 (0.8%) screened and found to be eligible of whom 121 (33.8%) signed informed consent.

Because of the low efficiency of recruiting frequency-matched controls using random-digit dialing, controls were subsequently recruited through address-based sampling using a list of addresses in the counties in the Philadelphia area from which TGCT cases were obtained. Potential control subjects were mailed an introductory letter describing the project and a screening questionnaire. Respondents who reported being male, without a personal history of TGCT, and between the ages of 18 and 55 years were sent an informed consent form and the research questionnaire. There were 67,750 attempted contacts with 1,821 (2.7%) screened and found to be eligible of which 1,021 (field response rate, 53.2%) signed informed consent. The overall response rate was 1.7%.

Our analytic dataset consists of 931 men without TGCT, which reflects additional exclusion criteria including residency outside of the nine Pennsylvania county area (n = 175), calculated age <18 or ≥55 years old (n = 33), individuals who withdrew from the study (n = 3), and individuals without available data (n = 1).

### Exclusions

Any participant reporting a genetic condition known to be associated with risk of TGCT, including Down syndrome and Klinefelter syndrome, and individuals with HIV, were excluded from all analyses.

### Variables

All study participants completed a self-administered questionnaire to elicit information on demographic data and known and presumptive risk factors for TGCT. Exposure to radiation was obtained through response to two primary questions: *Before your diagnosis of TGCT [men with TGCT]/index date [men without TGCT]*, *did you ever have an x-ray of your lower abdomen (below the waist) or a CT or CAT scan of the lower abdomen*? *Before your diagnosis of TGCT*, *did you ever have a lower GI series or barium enema*? When participants reported exposure to radiation they were subsequently asked to report the number of exposures and age at first exposure categorized as 0–10 years, 11–17 years, and ≥ 18 years; exact age at first exposure outside of these categories was not collected. Age was defined as the participant’s age at the last date of exposure ascertainment (date of diagnosis in men with TGCT and an index date specified in the questionnaire for men without TGCT). We refer to radiation exposure via any of the queried modalities, x-ray, CT or CAT scan, lower GI series, or barium enema, as “diagnostic radiation” throughout the remainder of the manuscript. Race was obtained by self-report.

### Statistical analysis

We compared demographic characteristics and known risk factors for TGCT between men with and without TGCT using a t-test for continuous and ordinal variables or a chi-squared test for categorical variables; and we similarly compared available demographic and tumor characteristics between participant and non-participant men with TGCT identified through the PACR. We examined 1) exposure to an x-ray or CT of the lower abdomen (below the waist); 2) exposure to a lower GI series or barium enema; and 3) exposure to x-ray, CT, or lower GI series or barium enema as a combined diagnostic radiation variable. We coded the number of exposures as 0, 1–2, and ≥ 3. We used multivariable logistic regression analyses to determine the independent risk of developing TGCT for the 1–2 and ≥ 3 exposure to diagnostic radiation groups using 0 exposures as the referent. We tested categories of exposures to diagnostic radiation for linear trend using the Mantel–Haenszel test, which we report as p for trend. We examined the association of demographic characteristics (age, race, county, year of birth, education), clinical variables (body mass index [BMI] at diagnosis), and known risk factors for TGCT (cryptorchidism, family history of TGCT) with the combined radiation exposure variable and utilized stepwise backward selection variable to determine a final adjustment model. All models were adjusted for age (continuous variable), year of birth (ordinal variable), race (binary variable: white versus all others given low counts among non-white participants) [18], family history of TGCT (binary variable), BMI at diagnosis (continuous variable), county (categorical variable with counties containing less than 10% of the proportion of the population collapsed into an “other” variable), and a history of cryptorchidism (binary variable). We report odds ratios (ORs) as a measure of risk along with corresponding 95% confidence intervals (CIs). All tests were two-sided and considered statistically significant when p-value < 0.05. We estimated population attributable risk (PAR), or the proportion of the incidence of TGCT in the population that may be due to diagnostic radiation, using the *regpar* function. We utilized multiple imputation with chained equations [19] to obtain imputed values for missing data from our self-report survey for the variables race (4%), cryptorchidism (2%), family history of TGCT (4%), BMI at diagnosis (1%) and exposure to diagnostic radiation (3%). All analyses were performed using Stata, version 15.0 (StataCorp).

## Results

There were 1,246 individuals meeting inclusion criteria including 315 men with TGCT and 931 men without TGCT. Men with TGCT who were enumerated in the PACR (2006–2010) who participated or did not participate in our study were similar in age, race, marital status, and tumor subtype (S1 Table in S1 File). The demographic characteristics and TGCT risk factors for all study participants are listed in Table 1. Among individuals with available pathology data (95%), 152 (51%) were seminomatous, 114 (38%) were non-seminomatous, and 34 (11%) were mixed.

**Table 1:** Characteristics of men with TGCT and men without TGCT.

	Men with TGCT (n = 315)	Men without TGCT (n = 931)	p-value
Age, mean (SD), years	34.7 (9.2)	40.3 (9.6)	<0.001
Year of birth, mean (SD)	1976 (9.6)	1971 (9.6)	<0.001
County			
Berks	12 (4)	76 (8)	
Bucks	49 (16)	130 (14)	
Chester	34 (11)	94 (10)	
Delaware	45 (14)	101 (11)	
Lancaster	17 (5)	134 (14)	<0.001
Lehigh	8 (3)	39 (4)	
Montgomery	67 (21)	156 (17)	
Northampton	5 (2)	39 (4)	
Philadelphia	78 (25)	162 (17)	
White, n (%)	301 (95)	846 (91)	0.026
College educated, n (%)	256 (81)	751 (81)	0.830
Family history of testicular cancer, n (%)	24 (8)	38 (4)	0.019
BMI at diagnosis, mean (SD), kg/m^2^	26.7 (4.4)	26.6 (4.6)	0.354
Cryptorchidism, n (%)	21 (7)	21 (2)	<0.001
Year of diagnosis, mean (SD)	2010 (2.3)	NA	NA

n, number; SD, standard deviation; NA, not applicable; BMI, body mass index.

The results for the association of categories of exposures to diagnostic radiation with TGCT are presented in Table 2. Exposure to any diagnostic radiation was reported in 28% of men with TGCT and 31% of men without TGCT. Compared to no exposure, there was a statistically significantly increased risk of TGCT with ≥3 exposures to x-ray or CT (OR = 1.78, 95% CI 1.15–2.76), lower GI series of barium enema (OR = 4.58, 95% CI 2.39–8.76), and the combined exposure variable (OR = 1.59, 95% CI 1.05–2.42). A trend of increasing risk with increasing category of exposure was only observed for lower GI series or barium enema (p<0.001) although it approached significance for exposure to x-ray or CT (p = 0.066).

**Table 2:** Risk of TGCT by exposure to diagnostic radiation.

Exposure	# of exposures	TGCT status (with/without)	OR (95% CI)	p-value	p for trend
X-ray or CT	0	235/699	ref	ref	
	1–2	38/148	0.82 (0.54–1.24)	0.338	0.066
	≥3	42/84	1.78 (1.15–2.76)	0.010	
Lower GI series or barium enema	0	265/792	ref	ref	
	1–2	25/115	0.92 (0.56–1.51)	0.742	<0.001
	≥3	25/24	4.58 (2.39–8.76)	<0.001	
X-ray, CT, lower GI series or barium enema	0	226/644	ref	ref	
	1–2	43/178	0.75 (0.50–1.11)	0.152	0.182
	≥3	46/109	1.59 (1.05–2.42)	0.029	

Adjusted for age, year of birth, race, history of cryptorchidism, family history of TGCT, county, and body mass index at diagnosis.

CI, confidence interval; CT, computed tomography; GI, gastrointestinal; OR, odds ratio.

The results for the association of age at first exposure to diagnostic radiation with TGCT are presented in Table 3. Compared to a first exposure after seventeen years of age, there was a non-statistically significantly increased risk of TGCT with first exposure at 0–10 years of age for x-ray or CT of the lower abdomen (OR = 2.17, 95% CI 0.86–5.50) and the combined variable (OR = 2.00, 95% CI 0.91–4.42).

**Table 3:** Risk of TGCT by age at first exposure to diagnostic radiation among individuals with at least one exposure to diagnostic radiation.

Exposure	Age at first exposure (years)	TGCT status (with/without)	OR (95% CI)	P-value	p for trend
X-ray or CT					
	≥ 18	46/169	ref	ref	0.184
	11–17	16/39	0.86 (0.36–2.07)	0.734	
	0–10	18/23	2.17 (0.86–5.50)	0.100	
Lower GI series or barium enema					
	≥ 18	36/113	ref	ref	0.974
	11–17	7/14	0.93 (0.21–4.11)	0.926	
	0–10	7/13	1.04 (0.25–4.40)	0.956	
X-ray, CT, lower GI series or barium enema					
	≥ 18	53/214	ref	ref	0.146
	11–17	18/44	0.91 (0.43–1.91)	0.795	
	0–10	18/29	2.00 (0.91–4.42)	0.086	

Adjusted for number of diagnostic radiation exposures, age, year of birth, race, history of cryptorchidism, family history of TGCT, county, and body mass index at diagnosis.

CI, confidence interval; CT, computed tomography; GI, gastrointestinal; OR, odds ratio.

Results from analyses using only observed data (S2 and S3 Tables in S1 File) were consistent with those using imputed values. We examined the population attributable risk by comparing the risk in a theoretical scenario in which no individual has three or more exposures to diagnostic radiation below the waist (and other covariates are distributed as in the observed data) to the scenario represented by our data. Removal of the exposure (i.e. 3+ exposures to diagnostic radiation), would prevent 1% of all TGCT from the population.

## Discussion

We found that individuals reporting a greater number of exposures to diagnostic radiation below the waist had an increased risk of TGCT. We also replicated known risk factors for TGCT including family history of TGCT and personal history of cryptorchidism. Our data suggest that increased utilization of diagnostic radiation could be contributing to a portion of the increasing incidence of TGCT.

The steadily increasing incidence of TGCT in recent decades [1] suggests an underlying prenatal or early age increasingly prevalent environmental exposure. However, no definitive risk factor robustly associated with the increasing TGCT incidence has been identified [2, 20]. Ionizing radiation is a risk factor for cancer due to its ability to induced DNA damage. When cells are unable to appropriately repair damaged DNA, cancer causing genetic mutations may result. Genome-wide association studies of TGCT have implicated regions containing genes encoding proteins involved in DNA damage response pathways including *SMARCAD1*, *RFWD3*, *RAD51C*, and *TERT* [21, 22]. Common variation associated with susceptibility to TGCT may confer sensitivity to DNA damage from ionizing radiation. Additionally, the use of diagnostic radiation, specifically the advent of CT scanning, and greater effective doses [8], has increased concurrently with TGCT incidence [3]. This trend is particularly true among primarily European descent countries where TGCT risk is the highest [3].

Epidemiologic studies on the association of ionizing radiation and TGCT are largely limited to occupational exposure. A review of 31 studies examining occupational exposure to radiation (e.g., nuclear and military workers) and TGCT risk found that some studies support an association, although the overall evidence for an association was limited [23]. Only one known study has examined the association between diagnostic radiation and TGCT [24]. Brown et al. conducted a case-control analysis in 271 men with TGCT and 259 men without TGCT to examine TGCT risk factors and found no association between x-rays below the waist and TGCT. However, few individuals in this small study likely underwent CT scanning, particularly at an early age, given that the study included TGCT diagnoses from 1976 to 1981.

Our finding that diagnostic radiation exposure may increase TGCT risk is supported by several prior observations. Diagnostic-radiation doses, in particular CT scanning, are above the dose thought to confer a clinically meaningful increased risk of solid malignancy [5, 15]. Although based on data from atomic bomb survivors, these data represent the best long-term quantitative assessment of low dose radiation induced cancer risk available [5], and recent studies support the association between CT scanning and cancer risk [14]. Our finding that an increased number of reported exposures to diagnostic radiation are potentially associated with a greater risk of TGCT is also supported by prior studies [6, 11–14]. Although not a statistically significant finding, our analyses point to a potential increased risk of TGCT with earlier age of exposure to diagnostic, consistent with the established relationship between earlier age of exposure and increased cancer risk [6]. However, these results should be interpreted with caution and investigated in larger cohorts.

We observed a non-statistically significantly decreased risk of TGCT among individuals reporting 1–2 versus no exposures to diagnostic radiation below the waist. Experimental and epidemiological evidence suggest that low doses of radiation could reduce cancer risk [25, 26]. However, the predominant model for cancer risk secondary to radiation exposure suggests a linear relationship with no minimum radiation threshold [5]. Our findings do not support a protective effect of diagnostic radiation on TGCT risk and the observed non-statistically significant decreased risk of TGCT with a limited number of exposures may be secondary to bias, such as selection bias, as discussed below.

This study has limitations. We utilized a case-control design, whereas a prospective study of the association between diagnostic radiation and TGCT would better for determining causality. However, a case-control design is a methodology that is ideally suited for studies investigating rare disease outcomes such as TGCT for which incidence approaches 6 in 100,000 men [27] and exposures with long latency periods like ionizing radiation (for solid tumors) [28]. A prospective study is probably infeasible due to the need for a very large sample size and long follow-up time.

Men with TGCT may be more likely to recall prior exposure to diagnostic radiation leading to biased results. Although recall bias is an inherent limitation in survey-based analyses [29], it is unlikely to completely explain our results. Exposure to diagnostic radiation is not an established risk factor for TGCT, which may mitigate the likelihood of differential recall [29]. It was not possible to validate self-reported exposure to diagnostic radiation, because we did not collect specific dates of exposure nor the names and locations of facilities where procedures occurred, and many individuals report remote histories of exposure (e.g. prior to 11 years of age). As UPHS is a tertiary health care system where patients are likely to receive some or all of their care elsewhere, a query of medical records for information on diagnostic radiation exposure prior to diagnosis of TGCT is not sensitive.

Selection bias, a known challenge to population-based research, is a concern in our study. To address this challenging limitation, we examined data from the PACR during the time period that we recruited men from the PACR (2006–2010). We were able to compare characteristics between men with TGCT enrolled in our study and men with TGCT not enrolled in our study, and we found no differences when comparing demographic and clinical variables (S1 Table in S1 File). The overall low response rate among control individuals further translates to a possible selection bias impacting our study results. We previously compared the characteristics of control individuals in our study to the National Health Interview Survey and found that our controls were similar to men in the Northeast [18]. However, we acknowledge that selection bias may impact the results of our study.

Our questionnaire was not written to distinguish between exposure to plain x-rays or CT scans below the waist. Considering this caveat, a reported dose delivered to the testes from a single exposure could range from <1 mSv [7] with a plain film to 20 mSv or higher with a CT scan [9]. A limited number of plain x-rays of the abdomen or pelvis, with an average effective dose of <1 mSv [7], may be unlikely to increase TGCT risk alone [6] and we are unable to investigate the relative contribution of plain x-rays of the abdomen or pelvis compared to CT scans in our analysis. Our questionnaire also did not ask participants about other non-TGCT malignancies and their related treatments. It is possible that some individuals may have had prior exposure radiation therapy related to non-testicular malignancies and we are unable to account for this in our analysis.

Although participants were solely asked about diagnostic imaging exposure prior to diagnosis, and x-ray based imaging below the waist is not part of the diagnostic work-up for TGCT [30], we cannot exclude the possibility that men with TGCT reported exposure to diagnostic radiation that was part of their post-diagnosis work-up for TGCT. Finally, we did not collect information on the dates of diagnostic radiation or exact age of first exposure beyond the described categories (0–11 years, 11–17 years, and ≥ 18 years), and we are therefore unable to determine the latency period between first diagnostic radiation exposure and TGCT diagnosis; this measure may have provided additional information regarding the interpretation of our results. Similarly, we are unable to exclude diagnostic radiation exposures immediately preceding TGCT diagnosis that would be unlikely to be causally associated with TGCT.

We demonstrate a possible association between reported exposure to diagnostic radiation and increased TGCT risk. Unlike other organs at risk for adverse effects from diagnostic radiation, the testes are rarely examined using x-ray based imaging directly and the opportunity for nearly complete shielding exists [15]. However, testicular shields are used appropriately in male children as little as 25% of the time [16]. If our results are validated, efforts to reduce medically unnecessary and avoidable testicular exposure should be considered, in part through efforts to reduce radiation dose and optimize shielding practices when appropriate. Ultimately, the benefits of diagnostic imaging are high relative to the risk of secondary malignancy.

## Supporting information

S1 File (XLSX)

S1 Data (CSV)
